# *NTRK* Fusions in Sarcomas: Diagnostic Challenges and Clinical Aspects

**DOI:** 10.3390/diagnostics11030478

**Published:** 2021-03-09

**Authors:** Vasiliki Siozopoulou, Evelien Smits, Koen De Winne, Elly Marcq, Patrick Pauwels

**Affiliations:** 1 Department of Pathology, Antwerp University Hospital, 2650 Edegem, Belgium; Koen.DeWinne@uza.be (K.D.W.); Patrick.pauwels@uza.be (P.P.); 2 Center for Oncological Research (CORE), Integrated Personalized and Precision Oncology Network (IPPON), University of Antwerp, 2610 Wilrijk, Belgium; Evelien.smits@uantwerpen.be (E.S.); Elly.Marcq@uantwerpen.be (E.M.); 3 Center for Cell Therapy and Regenerative Medicine, Antwerp University Hospital, 2650 Edegem, Belgium

**Keywords:** *NTRK*, sarcoma, histopathology, TRK-inhibitors, resistance, diagnostic algorithm

## Abstract

Tropomyosin receptor kinase (TK) is encoded by the neurotrophic tyrosine receptor kinase genes (*NTRK*) 1, 2, and 3, whose activation plays an important role in cell cycle proliferation and survival. Fusions of one of these genes can lead to constitutive activation of TRK, which can potentially be oncogenic. *NTRK* fusions are commonly present in rare histologic tumor types. Among sarcomas, infantile fibrosarcoma shows *NTRK* fusion in more than 90% of the cases. Many other sarcoma types are also investigated for *NTRK* fusions. These fusions are druggable alteration of the agnostic type, meaning that all *NTRK* fused tumors can be treated with NTRK-inhibitors regardless of tumor type or tissue of origin. TRK-inhibitors have shown good response rates, with durable effects and limited side effects. Resistance to therapy will eventually occur in some cases, wherefore the next-generation TRK-inhibitors are introduced. The diagnosis of *NTRK* fused tumors, among them sarcomas, remains an issue, as many algorithms but no guidelines exist to date. Given the importance of this diagnosis, in this paper we aim to (1) analyze the histopathological features of sarcomas that correlate more often with *NTRK* fusions, (2) give an overview of the TRK-inhibitors and the problems that arise from resistance to the therapy, and (3) discuss the diagnostic algorithms of *NTRK* fused tumors with emphasis on sarcomas.

## 1. Introduction

### The Role of NTRK in Oncogenesis

Tropomyosin receptor kinase (TRK) is a member of the tyrosine kinase family, predominantly expressed in neuronal tissue [1]. There are three receptors (TRK A, B, and C) encoded by the three neurotrophic tyrosine receptor kinase genes (*NTRK*), *NTRK* 1, 2, and 3, respectively. Activation of one of these three genes initiates a downstream signaling that impacts cell proliferation, differentiation, and survival. Normal/physiological TRK signaling is ligand dependent and plays a critical role in the nervous system development. TRK is expressed in some mature neural cells, such as ganglion cells. Due to intra- and inter-chromosomal translocations, *NTRK* can undergo chromosomal rearrangements resulting in gene fusions. This leads to ligand independent constitutive activation of the TRK pathway which can potentially be oncogenic [2].

*NTRK* fusions were first discovered in colon carcinoma in 1982 [3]. Regarding sarcomas, the *ETV6-NTRK3* fusion was described in congenital fibrosarcomas back in 1998 [4]. Nowadays, we recognize two major tumor categories based on the *NTRK* fusion frequency. In the first category, there are rare tumors with *NTRK* fusions in almost more than 90% of the cases [1], such as infantile fibrosarcoma, mammary analogue secretory carcinoma, secretory breast carcinoma, and congenital nephroblastic nephroma. The second category represents more common tumors with a low frequency for *NTRK* fusions, such as lung cancers, gastrointestinal cancers, melanoma, and thyroid cancers [1,2,5]. So far, more than 60 different fusion partners have been identified and many more novel fusions continue to emerge. *NTRK1* has got more fusion partners than *NTRK2* and *NTRK3* genes [6,7]. In general, though, *NTRK* fusions remain rare genetic events found in tumors. These gene fusions can represent targetable alterations. Targeting gene fusions is a paradigm of a tumor agnostic treatment, where the same drug is used for tumors with the same genetic alteration, regardless of the tumors site of origin or histologic type [8]. It is very important that all fusions can be targeted by the same class of drugs.

The Food and Drug Administration (FDA) approved the first-generation TRK-inhibitors, such as LOXO-101 (larotrectinib) [9] and entrectinib [10], for the treatment of tumors that harbor *NTRK* fusions. Nevertheless, additional mutations lead in some cases to resistance. Fortunately, the next-generation TRK-inhibitors, such as LOXO-195 [11] and TPX-0005 [12], have recently been introduced to overcome the resistance. TRK-inhibitors are in general well-tolerated drugs with good response rates in *NTRK* fused neoplasms. Hence, identification of patients that can benefit from this targeted therapy is of outmost importance. Till today, there are no pathognomonic morphological characteristics that correlate with the presence of *NTRK* fusions to be used as a diagnostic criterion. Nevertheless, in the various cases that have been published so far, some common features are described that could help us identify which tumors demand further investigation. The diagnosis still relies on the identification of *NTRK* fusion. Therefore, many algorithms have been introduced but no guidelines exist to date.

This paper gives an overview of the diagnostic challenges and the clinical importance of *NTRK* fusions in tumors, with special emphasis on sarcomas.

## 2. Histopathology of Sarcomas Showing *NTRK* Fusions

Sarcomas show a variety of morphological features, ranging from spindle cells to small round cells, epithelioid cells, histiocytoid cells etc. While reviewing the literature we found a range of case studies describing *NTRK* fused sarcomas. Among these, spindle cell tumors are the ones described to present *NTRK* fusions more often than other morphological types. Different growth patterns of *NTRK* fused spindle cell tumors have been analyzed in the literature, while researchers have often focused on the role of CD34 and S100 in these cases. Here we give an overview of the case studies found in the literature. Table 1 depicts all the fusion partners from the studies on soft tissue tumors listed below in correlation with the histomorphology.

One of the growth patterns that was described was the lipofibromatosis-like pattern. Lipofibromatosis (LPF) is known as a special type of benign mesenchymal tumors mostly arising in the pediatric population. Spindle cell tumors with an infiltrating growth pattern reminiscent of LPF, showing mild cytological atypia have been described to present recurrent *NTRK* fusions, especially of *NTRK1* [13,14]. Interestingly, because of—partial—CD34 and S100 protein immunohistochemical positivity, these cases were initially diagnosed as neural neoplasms. This tumor mostly affects the extremities of children and young adults. It is usually a low-grade, locally aggressive neoplasm with recurrences in cases of incomplete excision. Metastasis has only been reported in one case, but no fatalities have been recorded so far.

A case study describing a spindle cell neoplasm with myxoid features, low-grade morphology, and CD34 and partly S100 protein immunohistochemical positivity, had a rather aggressive course [15]. Some years after the initial diagnosis, the patient presented with metastatic disease in the lung and lymph nodes. An *LMNA-NTRK1* fusion was detected and a complete remission of the metastatic foci was achieved after one year of TRK-inhibitory therapy.

Fusions of the *NTRK1* gene also occur in low-grade sarcomas with a distinctive myopericytoma- or hemangiopericytoma-like morphology [16]. CD34 immunohistochemical positivity was not a consistent finding in these cases, while the S100 protein was not described. The tumors had a benign clinical course. The investigators of this study hypothesize that the presence of spindle cells with this distinctive growth pattern likely represents a novel morphologically and genetically defined soft tissue sarcoma (STS) entity with *NTRK1* fusions.

Spindle cell tumors of the gynecological tract, morphologically resembling fibrosarcomas can also harbor fusions of the *NTRK* [17,18,19], mainly of the *NTRK 1* and *3*. They are usually reported in younger patients, with the cervix being the predilection site. Largely varying morphological features and clinical outcomes are described. On the one hand, some of the tumors showed mild to moderate cytological atypia and in some instances a high mitotic activity. They all were CD34 and S100 protein positive. The tumors had a more aggressive course, nevertheless death from disease was not reported [17]. On the other hand, the tumors with high-grade morphology correspond to an aggressive disease that may, in some cases, be fatal. Interestingly, these tumor cells were negative for CD34 and only occasionally showed S100 protein positivity [18,19].

The majority of gastrointestinal stromal tumors (GISTs) are known to carry an oncogenic driver mutation, like KIT, PDGFRa, SDH, and BRAF mutations, which in most cases can be druggable. GISTs without one of those mutations are called “wild-type”. The investigators of the NCT02576431 trial performed a comprehensive genomic profile analysis of almost 190 “wild-type” GISTs, investigating genes that correspond to more than 300 somatic alterations [20]. An *ETV6-NTRK3* fusion was detected in two of the patients. Both were middle aged, males, with tumors located in the small and large intestine, respectively. The tumors showed no response when a conventional therapy was used. One of the patients was treated with larotrectinib and demonstrated ongoing partial response (PR) four months after initiation of the treatment. Other studies on “wild-type” spindle cell tumors of the gastrointestinal tract confirmed this finding [21,22]. A very interesting approach for the diagnosis of the *NTRK* fused gastrointestinal sarcomas is suggested by Atiq et al. [22]. They divided these tumors into two categories. The first category of *NTRK3* fused tumors includes high-grade infantile fibrosarcoma-like tumors. The second category of *NTRK1* fused tumors was further subdivided into low-grade CD34 and S100 protein positive tumors and into high-grade unclassified sarcomas.

*NTRK* fusions are also investigated in neoplasms with morphology resembling that of inflammatory myofibroblastic tumors (IMTs). Typically, IMT shows a characteristic spindle cell morphology with a prominent inflammatory infiltrate. Fifty percent of these cases display a clonal rearrangement involving the anaplastic lymphoma kinase (ALK) gene [23]. In addition, fusions of the ROS1 and PDGF-bèta genes also occur [24]. Recently, *ETV6-NTRK3* fusions have been described in three cases with IMT morphology that did not harbor an ALK rearrangement [25,26]. These tumors presented a striking plasma cell infiltrate. No CD34 and/or S100 protein positivity was described. One of the patients developed an advanced disease.

An *EML4-NTRK3* fusion was detected in a tumor with morphology suggestive of a dermatofibrosarcoma protuberans (DFSP) [27]. The tumor cells were positive for CD34, which is usually the case for DFSPs. In contrast, a COL1A1-PDGF-bèta fusion, typical of DFSPs, was not demonstrated in this case.

Another series presented two *NTRK3* fused fibrosarcoma-like spindle cell tumors of the extremities, one of which was located in the bone. The tumor cells were positive for CD34. Both cases were aggressive with the patients developing lung metastasis [28].

One of the largest studies investigating *NTRK* fusions in pediatric sarcomas included thirty patients [29]. An *ETV6-NTRK3* fusion was found in 40% of the cases, while the rest showed variable *NTRK* fusion partners. Morphologically, many different cytomorphological and architectural patterns were observed. All but two tumors showed a primitive spindle cell pattern and/or a fascicular/herringbone pattern. Myoid, IMT-like, and infiltrative/fibromatosis patterns were also described. Features included blood vessels with a hemangiopericytoma-like morphology as well as perivascular hyalinosis. Mitotic activity ranged from low to abundant while some tumors displayed necrosis. CD34 and/or S100 protein positivity was not detected in all cases. Recurrence rates were slightly higher for the non *ETV6*-positive tumors but the investigators mentioned that this was mainly due to incomplete excision of the lesions. Metastasis and death from disease sporadically occurred.

An attempt was made to divide the *NTRK1* fused spindle cell tumors into those with low and high cellular neoplasms [30]. CD34 and S100 protein was consistently expressed in both groups. Some of the low cellular neoplasms had keloidal stromal collagen deposition and perivascular hyalinized rings, or scattered pleomorphic and multinucleate tumor cells, or had an LPF-like morphology. This category of low cellular *NTRK1* fused tumors exhibited favorable prognosis. On the other hand, highly cellular tumors presented with pronounced atypia and showed a clear malignant behavior.

Similarly, the histopathological spectrum of NTRK3 fused spindle cell sarcomas has been extensively analyzed [31] and investigators divided them in two major categories. The first category contained tumors with low to intermediate cytologic atypia; mitotic activity could be brisk. Stromal hyalinization and perivascular collagen rings of an LPF-like morphology were described. The second category contained tumors reminiscent of fibrosarcoma or high-grade malignant peripheral nerve sheath tumor (MPNST). They exhibited high-grade cytomorphology with abundant mitotic features and sometimes necrosis. Notably, CD34 was positive in all cases as was the S100 protein in the majority of the cases. The investigators stress the fact that, in contrast to the low cellular *NTRK1* fused sarcomas, the low-intermediate grade *NTRK3* fused sarcomas can be aggressive neoplasms giving rise to metastatic disease.

In general, *NTRK1* fusions may be indolent, while *NTRK3* fusions mostly occur in aggressive tumors. Different histologic types have been analyzed, some of them presented more frequently. All tumors displayed a spindle cell morphology and the most common architectural patterns were LPF-like and fibrosarcoma-like. In addition, hemangiopericytoma-like and myxoid patterns were also described. Until now, no correlation between the fusion partner and the morphology or clinical outcome can be obtained.

Although a lot of research is done on soft tissue tumors, little is known about the role of NTRK fusions in bone sarcomas. A very interesting study revealed three osteosarcoma samples with non-functional *NTRK* fusions [32]. In the first case, an *NTRK2-UFD1* fusion was identified. This fusion led to a frame shift of the *NTRK2* resulting in a stop codon. Similarly, a premature stop codon occurred in the second case with an *NTRK3-VPS18* fusion. In the last case, the *NTRK3-RALGPS2* fusion displayed an in-frame start codon but this could not induce transcription of the functional domain of *NTRK3*. No morphology correlation was evaluated in these cases.

## 3. TRK-Inhibitory Therapy

### 3.1. Activity and Safety of the TRK Inhibitors in Sarcomas

Larotrectinib (LOXO-101) is one of the first FDA approved drugs for targeting tumors with *NTRK* fusions [9]. A multi-centric phase I study at the MD Anderson Cancer Center (NCT02122913) [33] investigated the dose escalation in seventy adult patients with locally advanced or metastatic solid tumor refractory to other standard treatment options. Among these were nine patients with STS and two patients with GIST. The patients were divided in six categories according to a standard 3 + 3 dose escalation scheme. Patients in cohort 1 received 50 mg larotrectinib once daily, in cohort 2100 mg once daily, in cohort 3 100 mg twice daily, in cohort 3a 200 mg once daily, in cohort 4150 mg twice daily and in cohort 5 they received 200 mg larotrectinib twice daily, in cycles of 28 days. The primary endpoint was drug safety and maximum tolerated dose (MTD) while secondary endpoints were pharmacokinetics, objective response (OR), and duration of response (DoR). The drug was generally well tolerated with sporadic grade 3 treatment-related adverse events (trAEs) but no grade 5 or 6 trAEs were seen. Investigators concluded that a dose of 100 mg twice daily correlates with the best MTD and duration of response.

Eight patients in this trial had an *NTRK* fusion proven by next-generation sequencing (NGS); six involved *NTRK3* and two *NTRK1*. An objective response rate (ORR) was seen in seven out of eight patients with *NTRK* fusions. Among the patients with fusions there was one patient with STS who showed PR and two patients with GIST, one with PR, and the other with complete response (CR). An acquired gatekeeper mutation (TRKC F617L) was detected in a patient with GIST after initial response to the treatment.

Another multi-centric, phase I study (NCT02637687) [34] aimed to investigate the safety of larotrectinib in pediatric tumors. Twenty-four children, adolescents, and young adults, age ranging from 1 month to 21 years, with locally advanced and metastatic disease were included. Patients were divided in three cohorts. In cohorts 1 and 2 doses were dependent on both age and bodyweight to achieve an area under the curve equivalent to the adult doses of 100 mg twice daily (cohort 1) and 150 mg twice daily (cohort 2). Cohort 3 included a dose of 100 mg/m^2^ twice daily (maximum 100 mg per dose); in contrast to the previous cohorts, dosage in this cohort was not dependent on age. Seventeen tumors were *NTRK* fused, among them fifteen sarcomas. Patients were divided in three cohorts and received different doses of larotrectinib. The secondary endpoints were MTD, ORR, progression free survival (PFS), and overall survival (OS). Patients tolerated larotrectinib well with only minor grade 3 trAEs. No grade 4 or 5 trAEs were observed. The recommended phase II dose was 100 mg/m^2^ body surface area twice daily in cycles of 28 days.

Among the twenty-four enrolled patients, twenty-two were available for response evaluation. Sixty-four percent of them showed an ORR, all of them displaying an *NTRK* fusion. Among the *NTRK* fused patients the ORR reached 93%, two with CR and twelve with PR. One patient with an acquired G623R solvent-front mutation developed resistance to the treatment.

Additional to its durable effects, larotrectinib demonstrated a rapid response onset. A newborn with an infantile fibrosarcoma of the tongue and extensive lymph node metastasis was treated with 100 mg/m^2^ body surface area of larotrectinib. Only two months after initiation of the therapy the patient showed a CR ongoing sixteen months, with negligible toxicity and no safety concerns [35].

Most of the clinical trials investigate TRK-inhibitors in locally advanced or metastatic solid tumors that are refractory to the standard treatment options. Larotrectinib has also been investigated in a neoadjuvant setting as a selective therapy targeting *NTRK* fusions to facilitate surgical resection in children with sarcoma [36]. This treatment is very promising especially in bulky, locally aggressive tumors since surgery can be mutilating.

Another important question that arises is whether TRK-inhibitory therapy should be given as a first line therapy or should be kept for cases that do not respond to the standard treatment. Given the general poor response of unclassified STSs to the standard chemotherapy, some suggest the introduction of TRK-inhibitors as first line treatment whenever the drug is accessible [37].

On the other hand, patients that receive TRK-inhibitors will eventually develop resistance, as will be discussed below. Therefore, some clinicians prefer to keep this treatment option only for cases refractory to the classical treatment protocols.

### 3.2. Resistance to TRK-Inhibitory Therapy

Despite the durable effects of TRK-inhibitors, an acquired resistance will eventually develop in some patients with *NTRK* fusions.

This resistance is mostly related to kinase domain mutations, namely solvent front or xDFG substitutions that cause structural changes at the ATP-binding site of the *NTRK*, reducing the ability of larotrectinib to adhere. A glycerine to arginine substitution in the solvent front of TRKA (G595R) and TRKC (G623R), as well as substitutions of the xDFG of TRKA (G667C) and TRKC (G696A), results in static clashes between the amino acids and components of larotrectinib. LOXO-195 and repotrectinib (TPX-0005), the two next-generation TRK-inhibitors, have been introduced in order to re-establish disease control and have already shown good results in patients with solvent front substitutions in clinical trials [11,12]. However, patients with acquired substitutions of the xDFG motif are more difficult to treat because they can show resistance to the next-generation TRK-inhibitors (type I). Very recently, a type II TRK-inhibitor was introduced, which along with the ATP pocket, also occupies the allosteric pocket that is accessible when the DFG motif is inactivated [38,39].

### 3.3. Non-Fusion NTRK Alterations and Their Role in Therapy

A variety of *NTRK* alterations, other than fusions, have been identified in several tumor types: point mutations, amplifications, deletions, and splice variants [40]. Nevertheless, to date very limited response has been established in tumors with non-fused *NTRK* alterations treated with larotrectinib. Hong et al. performed a meta-analysis of three clinical trials (NCT02122913, NCT02576431, NCT02637687) and showed that among the seventy-three patients that did not harbor an *NTRK* fusion, eight had an *NTRK* point mutation, seven an *NTRK* amplification, four an *NTRK* rearrangement, and one had an *NTRK* deletion [41]. Only one patient with an *NTRK* amplification presented a PR of short duration. None of the patients with a point mutation responded to the therapy with larotrectinib. In a case presentation, a metastatic esophageal squamous cell carcinoma with an *NTRK1* amplification that was treated with larotrectinib reported a PR of the primary and metastatic foci; yet, 3.5 months after the treatment initiation, the patient exhibited disease progression [42].

### 3.4. Additional Genetic Mechanisms in NTRK Sarcomas

According to the NGS data from the clinical trial of larotrectinib in *NTRK* fused tumors (NCT02122913), patients with such fusions showed no other actionable alterations within the same tumor [33]. Moreover, all tumors described previously harboring an *NTRK* fusion, lacked another oncogenic driver mechanism. This implies that sarcomas with a known oncogenic driver event will not harbor *NTRK* fusions. Testing for *NTRK* fusions may not be applicable for specific histological types with a known or proven oncogenic driver mechanism, like synovial sarcoma or alveolar rhabdomyosarcoma.

In some cases of *NTRK1* and *NTRK3* fused tumors, an additional secondary cyclin-dependent kinase inhibitor 2A (CDKN2A) deletion has been described [16,17,28,31,43]. The CDKN2A tumor-suppressor locus on chromosome band 9p21 can be inactivated, and this locus is known to be prone to homozygous deletions in a wide range of human cancers [44]. The significance of this finding is unclear. However, it is suggested that especially for the *NTRK1* fusions an additional genetic mechanism is needed in order to induce carcinogenesis [16]. One of the cases with CDKN2A deletion also showed a simultaneous p53 mutation [31]. Finally, non-random gains in one or multiple chromosomes (8, 11, 17, and 20) previously associated with infantile fibrosarcoma have also been mentioned [29]. A rare case with an *NTRK1* fusion showed amplification of the fusion gene locus [16].

## 4. Diagnostic Algorithms

### 4.1. Techniques to Identify NTRK Fused Sarcomas

As illustrated, patients with sarcomas that harbor *NTRK* fusion are good candidates for TRK-inhibitory therapy in adjuvant, neoadjuvant, or metastatic setting. The response rates are durable and the drugs are well tolerated, even in younger ages. Resistance to the first-generation inhibitors can be largely tackled by introducing the new generation inhibitors. Therefore, identification of the *NTRK* fused malignancies is of outmost importance.

**Figure 1:**
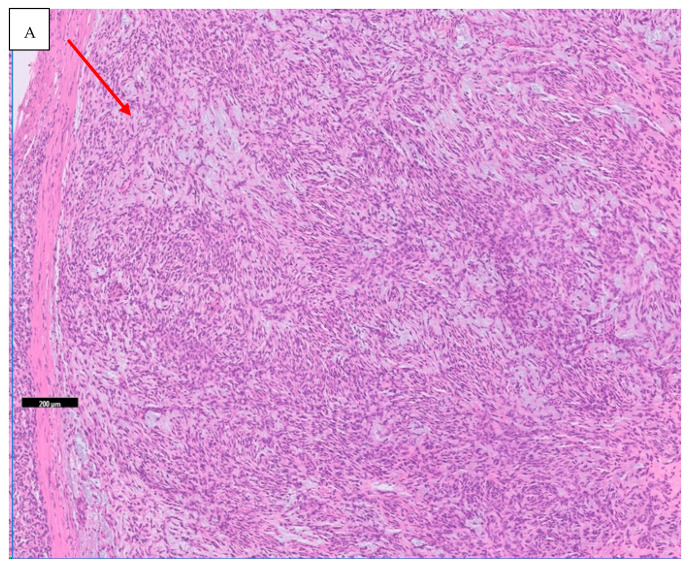

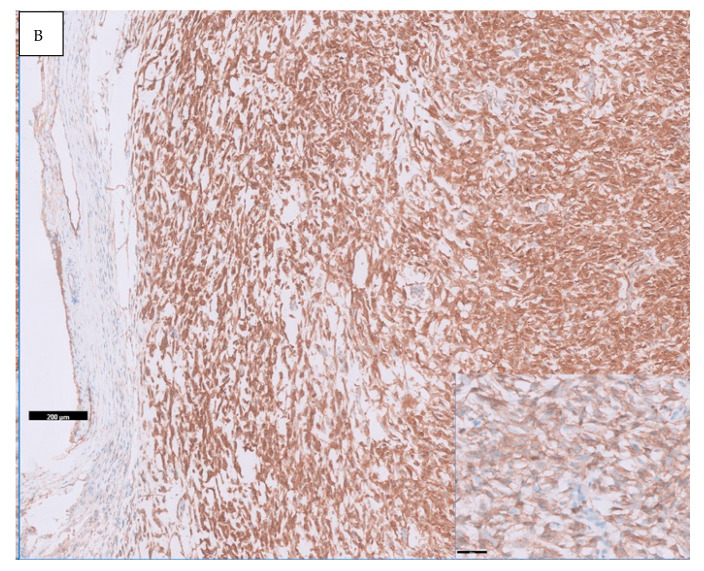
Spindle cell tumor on the deep soft tissue of the back with myxoid stroma (arrow) and diffuse cytoplasmatic positivity for pan-TRK IHC (**A**: Hematoxyline-Eosine, scale bar 200 μm, **B**: Antibody: EPR17341, Roche; Chromogene: DAB, scale bar 200 μm, insertion 50 μm).

Morphology can augment in selecting those tumor types that are more likely to display an *NTRK* fusion. Immunohistochemistry reveals protein expression and has been proposed as a method to pre-screen for the presence of *NTRK* fusions [45]. Figure 1 shows a spindle cell tumor with diffuse positivity for pan-TRK immunohistochemistry.

There are different pan-TRK immunohistochemical antibodies such as the EPR17341 from Roche and the A7H6R from Cell Signaling Technology. The sensitivity of this technique is not optimal, especially for detecting the *NTRK3*, which according to different studies does not exceed 79% [46,47]. It has been proven that samples from tumors with an amplification, mainly of the *NTRK1*, can show TRK immunohistochemical positivity in nearly 15% of the cases [48]. Hence, TRK immunohistochemical positivity can correlate also to other, non-fusion alterations of the *NTRK*. Although the technique lacks specified criteria for evaluation, a Belgian Ring Trial for pan-TRK immunohistochemistry showed excellent concordance between the participating laboratories compared to the referral centrum [49].

Confirmation of the fusion comes through molecular techniques. This can happen through Fluorescence in Situ Hybridization (FISH), although it does require using the three FISH probes for *NTRK1*, *2*, and *3* separately [50]. Real Time Polymerase Chain Reaction (RT-PCR) is also readily available and a low-cost procedure. However, as the fusion partner has to be known, it cannot detect any novel fusions [51,52]. DNA-based sequencing can be problematic because *NTRK*, especially *NTRK2* and *NTRK3*, have large intronic regions in which all breakpoints cannot be adequately covered [53]. Instead, RNA-based sequencing is more preferable, given that splicing out of introns simplifies the technical requirements [54]. For RNA-based NGS, the quality of the sample plays a critical role [50]. This is particularly crucial in cases of bone sarcomas, where fixation and demineralization procedures can result in RNA degradation [55].

### 4.2. Guidelines and Algorithms for Identification of NTRK Fused Sarcomas

For patients to benefit from a targeted therapy, it is essential to identify tumors with *NTRK* fusions. Until today, different testing algorithms have been proposed but no guidelines exist. There are three main algorithms published to date; those proposed by the ESMO [54], those from the Memorial Sloan Kettering Cancer Center [56], and those presented by Penault-Liorca et al. [57]. In addition, the Canadian Consensus has published its own data recently [37]. Despite the differences between these algorithms, the principle remains the same. Regarding sarcomas, in case that the tumor is known to have a high prevalence of *NTRK* fusions (e.g., infantile fibrosarcoma), direct confirmation with molecular techniques is recommended. If not, one can choose to perform immunohistochemistry with a pan-TRK antibody as an enrichment strategy. In cases that immunohistochemistry reveals an NTRK protein expression, a confirmation with molecular techniques is necessary. The molecular technique favored in most cases is the NGS at RNA level.

**Scheme 1:**
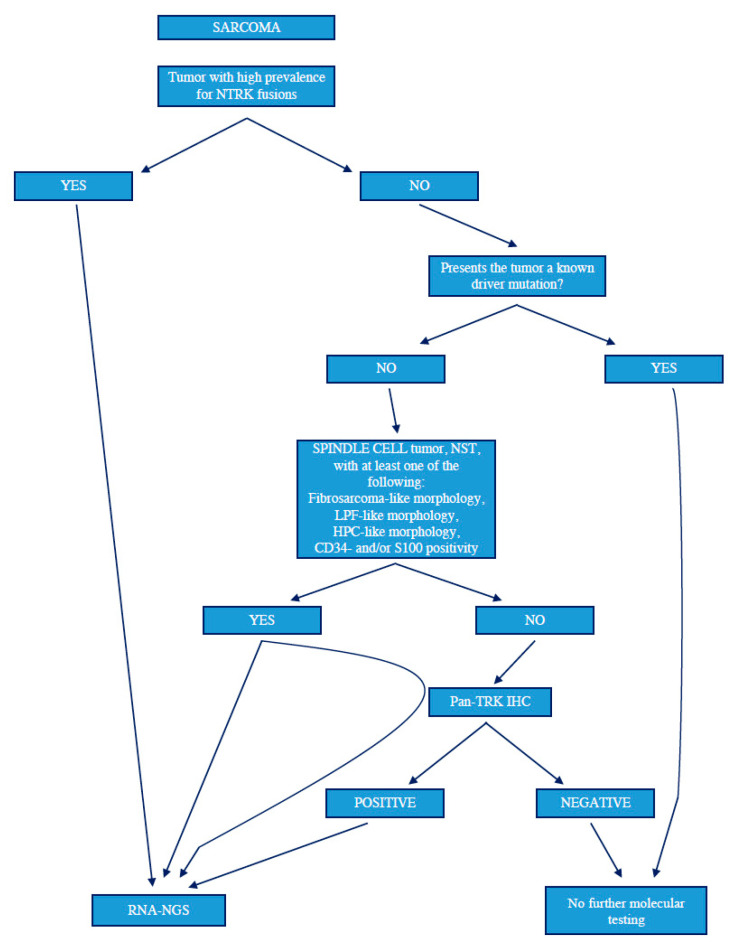
Diagnostic algorithm for the identification of NTRK fused sarcomas, according to the data presented in this review. In cases of a spindle cell tumor NST with a histologic profile suggestive for a *NTRK* fusion, one can proceed directly to molecular testing or perform a pan-TRK IHC together with molecular testing, the latter in order to investigate the sensitivity of IHC especially in cases of NTRK3 gene fusions. The preferable molecular technique is RNA-NGS. Abbreviations: HPC; hemangiopericytoma IHC; immunohistochemistry; LPF; lipofibromatosis, NGS; Next Generation Sequencing, NST; non special type.

To date, each laboratory uses its individual strategy for the identification of the *NTRK* fused sarcomas and other tumors, therefore no suggestion can be made about the effectiveness of each diagnostic algorithm. Scheme 1 suggests a diagnostic algorithm for detection of *NTRK* fusions in sarcomas, according to the data presented in this review.

## 5. Conclusions

The presence of an *NTRK* fusion is a rare genetic event arising in sarcomas. Inhibition of TRK is a promising and well-tolerated targeted therapy for selective tumors. An additional advantage of this treatment approach is that the same drug targets all three types of *NTRK* fusions. Today, most of the data show that all *NTRK* fused tumors are highly responsive to the available TRK-inhibitors. The efficacy of those drugs in sarcomas with *NTRK* alterations other than fusion remains questionable. Occasionally, patients with *NTRK* amplified tumors may respond to TRK-inhibitors, still this effect might not be durable. Hence, it is clear that standardization of the diagnostic algorithm is of outmost importance in order to select the patient population that is more likely to benefit from the therapy.

The histological picture seems to play an essential role in the identification of sarcomas that are good candidates for immunohistochemical and/or molecular testing. *NTRK* fusions are oncogenic driver alterations, therefore, tumors with a known driver alteration (mutation, translocation, amplification) are very unlikely to harbor *NTRK* fusions. As a consequence, molecular testing for those tumors may not be applicable. In summary, spindle cell tumors with a fibrosarcoma-, LPF-, myopericytoma/hemangiopericytoma-like or myxoid morphological pattern, positive for CD34 and/or S100 protein, but without a known driver oncogenic alteration, meet the suggested criteria for NTRK fusion testing. Confirmation warrants molecular techniques, with NGS testing at RNA level being the most preferable.

To date, *NTRK* fusions in osteosarcomas do not seem to be functional alterations. Nevertheless, given the limited data that are available, further investigation is needed.

Although TRK is a promising target for therapy with durable effects, resistance to first-generation drugs has already made its appearance. Next-generation drugs can re-establish disease control, still this is a considerable obstacle leading to uncertainty about the long-term effectiveness of this therapy.

## Figures and Tables

**Table 1:** Correlation between the different neurotrophic tyrosine receptor kinase genes (*NTRK*) fusions and main histo-morphological features of the soft tissue tumors as described in different publications.

*NTRK*	Fusion Partner	Different Publication Describing Histo-Morphological Features in Soft Tissue Sarcomas, in Regard to Different Fusion Partners														
		Kao et al. [13]	Agaram et al. [14]	So et al. [15]	Haller et al. [16]	Croce et al. [17]	Chiang et al. [18]	Rabban et al. [19]	Shi et al. [20] Brenca et al. [21]	Atiq et al. [22]	Alassiri et al. [25] Yamamoto et al. [26]	Olson et al. [27]	Yamazaki et al. [28]	Davis et al. [29]	Suurmeijer et al. [30]	Suurmeijer et al. [31]
*NTRK1*	*TPR*	LPF-like	LC SCT				Uterine/cervix; FS-like	Cervix; HC SCT Adenosarcoma-like		«Wild type» GIST: HG				IFS-like	LC SCT	
*NTRK1*	*TPM3*	LPF-like	LC SCT HC SCT		MFS-like MPC-like	Cervix; FS-like	Uterine/cervix; FS-like	Cervix; HC SCT adenosarcoma-like		«Wild type» GIST: IFS-like or HG				IFS, LG SCT, Unclassified, Inflammatory SCT/RCT, Inflammatory fibroid polyp	LC SCT SCT with increased cellularity	
*NTRK1*	*LMNA*	LPF-like	LC SCT HC SCT	SCT with myxoid features	Infantile HPC-like		Uterine/cervix; FS-like			«Wild type» GIST DFSP-like				Myxoid DFSP-, IFS-, Cellular schwannoma-like	LC SCT SCT with increased cellularity	
*NTRK1*	*MIR584F1*													IFS-like		
*NTRK2*	*SQSTM1*													Unclassified		
*NTRK3 NTRK2*	*STRN*												FS-like (NTRK3)	Unclassified (NTRK2)		
*NTRK3*	*SPECC1L*	LPF-like (NTRK2)	LC SCT (NTRK2)					Cervix; HC SCT adenosarcoma-like (NTRK3)		«Wild type» GIST: IFS-like appearance (NTRK3)					LC SCT: perivascular thick collagen & LPS-like component (NTRK2)	
*NTRK3*	*TFG*	LPF-like												LG SCT		IG SCT
*NTRK3*	*EML4*					Cervix; FS-like						DFSP-like				HG SCT
*NTRK3*	*RBPMS*						Uterine/cervix; FS-like							Unclassified, SCT, RCT, IFS, LG SCT		
*NTRK3*	*ETV6*								«Wild type» GIST-like	«Wild type» GIST: IFS-like	IMT-like					IG SCT
*NTRK3*	*STRN3*												FS-like			
	*TMP4*															HG SCT

Abbreviations: DFSP; dermatofibrosarcoma protuberans, FS; fibrosarcoma, GIST; gastrointestinal stromal tumor, HC; high cellular/high cellularity, HG; high grade, HPC; hemangiopericytoma, IFS; infantile fibrosarcoma, IG; intermediate grade, IMT; Inflammatory Myofibroblastic Tumor, MFS; myofibrosarcoma, MPC; myopericytoma, LC; Low cellular/low cellularity, LPF; lipofibromatosis, LG; low grade, RCT; round cell tumor, SCT; spindle cell tumor.

## Data Availability

Not applicable.
